# UFR2709, a Nicotinic Acetylcholine Receptor Antagonist, Decreases Ethanol Intake in Alcohol-Preferring Rats

**DOI:** 10.3389/fphar.2019.01429

**Published:** 2019-12-03

**Authors:** Gabriel Quiroz, Ramón Sotomayor-Zárate, Juan Pablo González-Gutierrez, Franco Vizcarra, Felipe Moraga, Isabel Bermudez, Miguel Reyes-Parada, María Elena Quintanilla, Diego Lagos, Mario Rivera-Meza, Patricio Iturriaga-Vásquez

**Affiliations:** ^1^Programa de Doctorado en Farmacología, Facultad de Ciencias Químicas y Farmacéuticas, Universidad de Chile, Santiago, Chile; ^2^Laboratorio de Neuroquímica y Neurofarmacología, Centro de Neurobiología y Fisiopatología Integrativa (CENFI), Instituto de Fisiología, Facultad de Ciencias, Universidad de Valparaíso, Valparaíso, Chile; ^3^Programa de Doctorado en Química, Facultad de Ciencias, Universidad de Chile, Santiago, Chile; ^4^Laboratorio de Síntesis Orgánica y Farmacología Molecular, Departamento de Ciencias Químicas y Recursos Naturales, Facultad de Ingeniería y Ciencias, Universidad de la Frontera, Temuco, Chile; ^5^Deptartment of Biological & Medical Sciences, Faculty of Health & Life Sciences, Oxford Brookes University, Oxford, United Kingdom; ^6^Centro de Investigación Biomédica y Aplicada (CIBAP), Escuela de Medicina, Facultad de Ciencias Médicas, Universidad de Santiago de Chile, Santiago, Chile; ^7^Facultad de Ciencias de la Salud, Universidad Autónoma de Chile, Santiago, Chile; ^8^Programa de Farmacología Molecular y Clínica, ICBM, Facultad de Medicina, Universidad de Chile, Santiago, Chile; ^9^Departamento de Química Farmacológica y Toxicológica, Facultad de Ciencias Químicas y Farmacéuticas, Universidad de Chile, Santiago, Chile; ^10^Center of Excellence in Biotechnology Research Applied to the Environment, Universidad de La Frontera, Temuco, Chile

**Keywords:** alcohol dependence, ethanol, UChB rats, nAChR antagonism, voluntary ethanol drinking

## Abstract

Brain nicotinic acetylcholine receptors (nAChRs), a heterogeneous family of pentameric acetylcholine-gated cation channels, have been suggested as molecular targets for the treatment of alcohol abuse and dependence. Here, we examined the effect of the competitive nAChR antagonist UFR2709 on the alcohol consumption of high-alcohol-drinking UChB rats. UChB rats were given free access to ethanol for 24-h periods in a two-bottle free choice paradigm and their ethanol and water intake were measured. The animals were i.p. injected daily for 17 days with a 10, 5, 2.5, or 1 mg/kg dose of UFR2709. Potential confounding motor effects of UFR2709 were assessed by examining the locomotor activity of animals administered the highest dose of UR2709 tested (10 mg/kg i.p.). UFR2709 reduced ethanol consumption and ethanol preference and increased water consumption in a dose-dependent manner. The most effective dose of UFR2709 was 2.5 mg/kg, which induced a 56% reduction in alcohol consumption. Administration of UFR2709 did not affect the weight or locomotor activity of the rats, suggesting that its effects on alcohol consumption and preference were mediated by specific nAChRs.

## Introduction

Alcohol is the most commonly abused legal substance and alcoholism is a serious public health problem worldwide (WHO, 2014). Several lines of evidence have identified neuronal nicotinic acetylcholine receptors (nAChRs) in the mesocorticolimbic-dopamine (DA) system as being involved in alcoholism (Madden and Heath, 2002; John et al., 2003; Dani and Harris, 2005; Falk et al., 2006). Consistent with this view, nAChR ligands reduce ethanol consumption in various animal models and humans (Chatterjee and Bartlett, 2010; Rahman et al., 2015).

nAChRs belong to the pentameric ligand-gated ion channel superfamily. The most abundant nAChRs in the brain are the heteromeric α4β2 and homomeric α7 subtypes (Gotti et al., 2006; Albuquerque et al., 2009). Other heteromeric nAChRs present in the brain include the α4β2α5, α6β2β3, α4β2α6, α4β4, α3β4 and α3β2 subtypes, but these are less abundant and/or have a more restricted distribution (Gotti et al., 2006). Ethanol intake appears to involve a variety of nAChR subtypes (Joslyn et al., 2008; Saccone et al., 2009; Taslim and Saeed Dar, 2011). α7 (Kamens et al., 2010a) and α3β4 nAChRs (Chatterjee et al., 2011; Miller et al., 2019) have both been implicated in ethanol intake, and receptors containing the α5 nAChR subunit are thought to be associated with the sedative effects of ethanol (Santos et al., 2013). Furthermore, the α4β2 nAChR subtype may be involved in alcohol intake due to its role in the brain’s reward system. Ethanol activates the mesolimbic-DA system, inducing the release of DA in the nucleus accumbens from projections that arise in the ventral tegmental area (VTA) (Gessa et al., 1985; Di Chiara and Imperato, 1988). VTA dopaminergic neurons primarily express the α4β2 nAChR subtype, but also express nAChRs exhibiting combinations of α5 and α6 subunits (Klink et al, 2001; Azam et al., 2002). These nAChRs are activated by cholinergic inputs from the laterodorsal tegmental and pedunculopontine tegmental nuclei (Oakman et al., 1995; Jerlhag et al., 2012; Xiao et al., 2016). Interestingly, voluntary ethanol consumption increases ACh levels in the VTA and promotes DA overflow in the nucleus accumbens in rats (Larsson et al., 2005). This establishes a cholinergic-dopaminergic reward axis (Xiao et al., 2016), which is affected by ethanol (Engel and Jerlhag, 2014).

Partial agonists of nAChRs such as cytisine (Papke and Heinemann, 1993; Rollema et al., 2010) and varenicline (Coe et al., 2005; Rollema et al., 2007) have been shown to decrease ethanol intake in rodents after a single dose or short-term administration (Steensland et al., 2007; Kamens et al., 2010b; Sajja and Rahman, 2011). Indeed, we confirmed the effects of varenicline and cytisine on alcohol intake in alcohol-preferring University of Chile (UChB) rats (Sotomayor-Zárate et al., 2013). These animals have been selectively bred for over 90 generations for their ethanol preference and are considered suitable models of alcohol dependence (Mardones and Segovia-Riquelme, 1983; Quintanilla et al., 2006; Tampier and Quintanilla, 2010). Given that partial agonists display competitive antagonistic effects in the presence of a full agonist, the effects of cytisine and varenicline on alcohol intake may stem from their antagonistic effects rather than from their partial activation of nAChRs. Consistent with this possibility, we showed that administration of erysodine, a competitive nAChR inhibitor, induced a marked decrease in alcohol intake in UChB rats (Quiroz et al., 2018). To further explore the efficacy of nAChR inhibitors in reducing alcohol intake, we assessed the effects of UFR2709 [(S)-1-methylpyrrolidin-2-yl) methyl benzoate], a recently described competitive nAChR antagonist (Faundez-Parraguez et al., 2013), on the maintenance of ethanol intake by alcohol-preferring UChB rats. Here, we show that UFR2709 reduces ethanol intake in a dose-dependent manner without affecting body weight or locomotor activity.

## Materials and Methods

### Drugs and Drinking Solutions

UFR2709-HCl (M.W. 255.74 g/mol) was synthesized as previously reported (Faundez-Parraguez et al., 2013). The structure of UFR2709-HCl was confirmed by one- and two-dimensional ^1^H and ^13^C NMR analyses. Nicotine ditartrate was purchased from Sigma-Aldrich (St. Louis, MO, USA). All other reagents used were of analytical grade. The volume of injection (1 ml/kg) was adjusted to body weight to achieve the desired dose of UFR2709-HCl. Ethanol solutions (10% ^v^/_v_) were prepared by mixing absolute ethanol (Merck, Darmstadt, Germany) with tap water. Ethanol concentration was chosen based on prior studies using UChB rats (Mardones and Segovia-Riquelme, 1983; Quintanilla et al., 2006).

### Animals

The experiments were carried out in male Wistar-UChB rats (n = 37). The UChB rat line has been bred for over 90 generations to ingest 10% ethanol solution in preference to water (Mardones and Segovia-Riquelme, 1983; Quintanilla et al., 2006). Thus, these animals are considered suitable models of alcoholism and are used to screen medications to treat alcoholism (Quintanilla et al., 2006). UChB rats weighing between 240 and 280 g were housed individually (for ethanol consumption experiments) or in trios (for locomotor activity experiments) in polycarbonate cages in temperature- and humidity-controlled conditions under a regular 12-h light-dark cycle (lights off at 19:00 h) with free access to food and water. All alcohol consumption experiments were performed at the Faculty of Medicine, Universidad de Chile.

All animal experiments were performed in accordance with ARRIVE guidelines (Kilkenny et al., 2010) and approved by the “Animal Experimentation Ethics Committee of the Universidad de Chile”.

### Effect of Different Doses of UFR2709 on the Maintenance of Ethanol Intake by UChB Rats

The ethanol preference of UChB rats administered different doses of UFR2709 was assessed using a two-bottle free choice experimental paradigm, as previously described (Mardones and Segovia-Riquelme, 1983; Quintanilla et al., 2006; Tampier and Quintanilla, 2010; Sotomayor-Zárate et al., 2013). Twenty-five UChB rats were housed in individual cages and subjected to a homecage two-bottle free choice regimen between ethanol 10% ^v^/_v_ and distilled water with continuous access (24 h/day). The positions of the bottles were alternated daily to avoid potential position preference. After 20 days, a stable plateau of ethanol consumption was reached, and the final three drinking days were averaged to obtain the mean voluntary ethanol consumption of each rat. The rats were then randomly divided into five groups (n = 5 per group), and a single i.p. injection of UFR2709-HCl (1, 2.5, 5, or 10 mg/kg) or saline was administered for 17 days at 15:00 h. After the treatment period, all UChB rats were maintained under the 24-h continuous access two-bottle free choice paradigm for three additional days. The animals were allowed *ad libitum* access to food. The weight and ethanol and water intake of the animals were recorded at 14:00 h each day and expressed as g ethanol/kg/day and mL water/kg/day, respectively.

### Effect of UFR2709 on Locomotor Activity

Locomotor activity was assessed using the open-field test, as previously described (Rivera-Meza et al., 2014). The open-field apparatus consisted of a black polycarbonate chamber (43 × 43 × 43 cm), the floor of which was marked with lines (length: 14.3 cm) forming a 3 × 3 grid. To study the effects of UFR2709 on locomotor activity, 12 ethanol-naïve UChB rats were randomly assigned to two groups and administered a 10 mg/kg dose (i.p.) of UFR2709 (n = 6) or an equivalent volume of saline (n = 6) (1 mL/kg). After 30 min of UFR2709 or saline administration, the animals were individually placed in the center of the open-field apparatus, and their locomotor activity was recorded for 30 min. Locomotor activity was recorded by a digital camera which was fixed above the test chamber and connected to a computer in another room. The apparatus was wiped and cleaned with water after each trial. Horizontal locomotor activity was expressed as activity units (AUs) per 5 min. An AU was defined as complete crossing from one square to another. The number of times of vertical rear per 5 min and the time (in s) spent in grooming behavior were also measured.

### Determination of Octanol-Buffer Distribution Coefficient of UFR2709 At pH 7.4

Octanol-buffer distribution coefficient at pH 7.4 (Log D_7.4_) values were determined using the shake-flask method (Andrés et al., 2015). Briefly, 5 mg of UFR2709-HCl and nicotine were added to 5 mL of 50 mM phosphate buffer (pH 7.4) and 5 mL of n-octanol (water saturated) in a glass vial. The sample vial was mixed by vortexing and then incubated to equilibrium for 24 h at 25°C. After equilibration, the phases were separated and the compounds were measured by UV spectroscopy at a wavelength of 232 nm for UFR2709-HCl and 257 nm for nicotine using calibration curves. The logarithm of the quotient of the concentrations in the organic and aqueous phases (Log D_7.4_) was calculated. Values correspond to the mean ± SEM of five independent assays.

### Statistical Analysis

**Figure 1:**
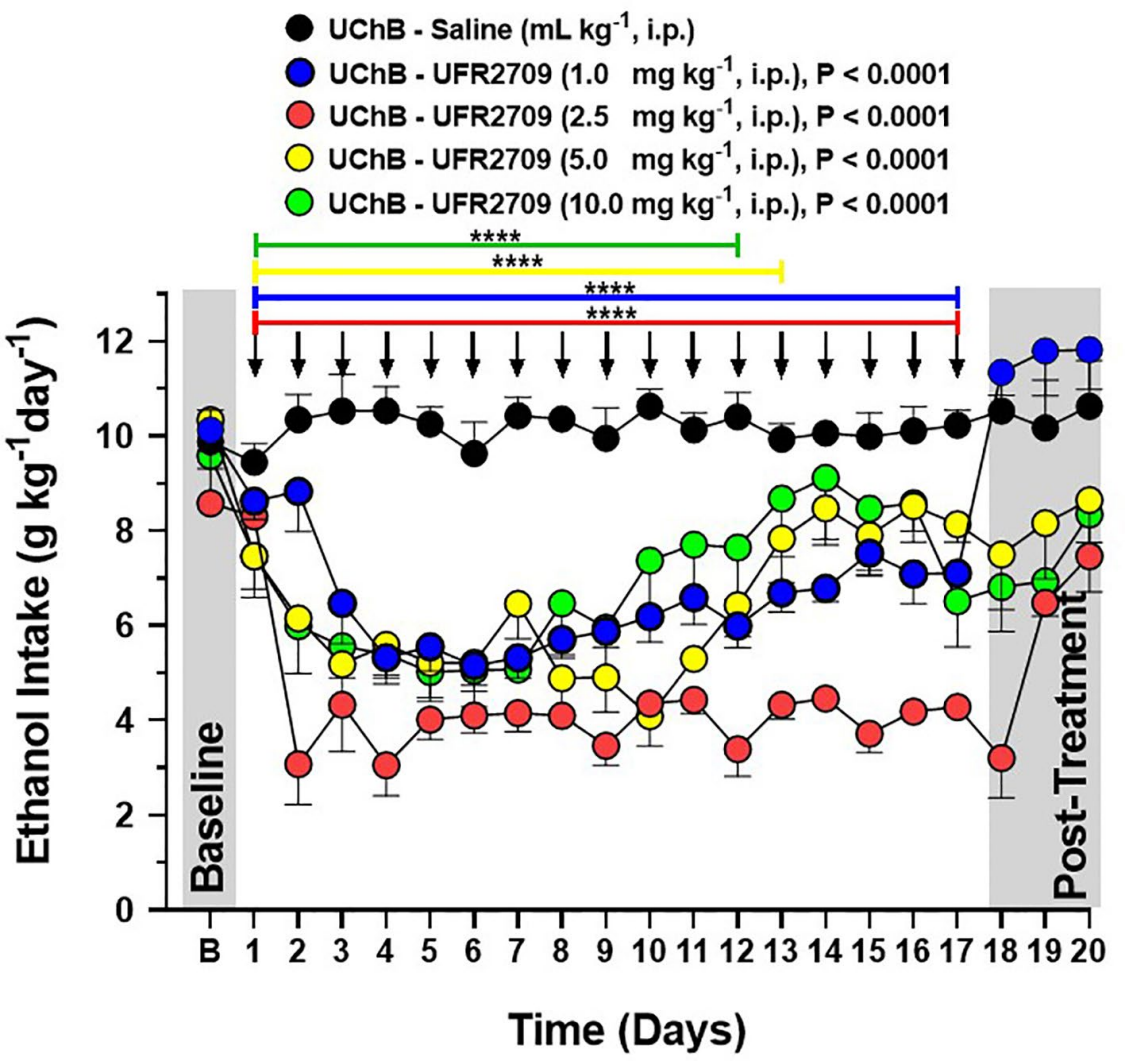
Influence of 17 days of UFR2709 treatment on the voluntary ethanol intake of high-alcohol-drinking UChB rats under a 24-h access two-bottle free choice paradigm. The baseline ethanol consumption of each experimental group is the average ethanol intake during the last 3 days before the treatment period. For 17 consecutive days, rats (n = 5 per group) were administered a single i.p. injection of UFR2709 (1, 2.5, 5, or 10 mg/kg/day) or saline (1 mL/kg/day) at 15:00 h, and ethanol consumption was recorded at 14:00 h the next day. Ethanol consumption data are expressed as mean ± SEM (g/kg/day). Two-way ANOVA with Tukey’s multiple comparison test was used to analyze the effect of UFR2709 treatment on ethanol consumption (P < 0.0001). Arrows indicate the time points of UFR2709 (1, 2.5, 5, or 10 mg/kg) or saline (1 mL/kg) administration *via* i.p. injection. ****0.0001.

**Figure 2:**
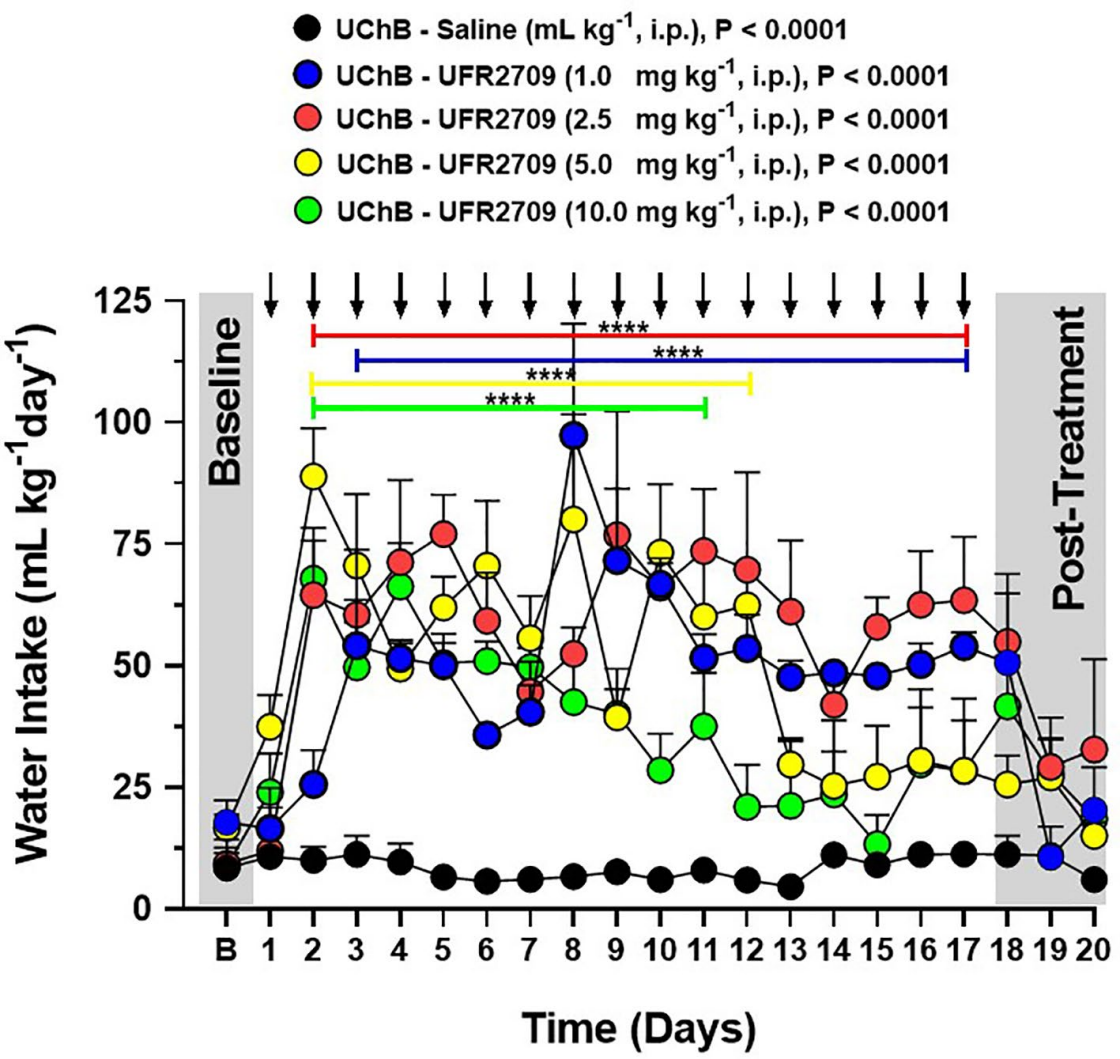
Influence of 17 days of UFR2709 treatment on the water intake of high-alcohol-drinking UChB rats under a 24-h access two-bottle free choice paradigm. The baseline water consumption of each experimental group is the average water intake during the last three days before the treatment period. For 17 consecutive days, rats (n = 5 per group) were administered a single i.p. injection of UFR2709 (1, 2.5, 5, or 10 mg/kg/day) or saline (1 mL/kg/day) at 15:00 h, and water intake was recorded at 14:00 h the next day. Water intake data are expressed as mean ± SEM (mL/kg/day). Two-way ANOVA with Tukey’s multiple comparison test was used to analyze the effect of UFR2709 treatment on water intake (P < 0.0001). Arrows indicate the time points of UFR2709 (1, 2.5, 5, or 10 mg/kg) or saline (1 mL/kg) administration *via* i.p. injection. ****0.0001.

**Figure 3:**
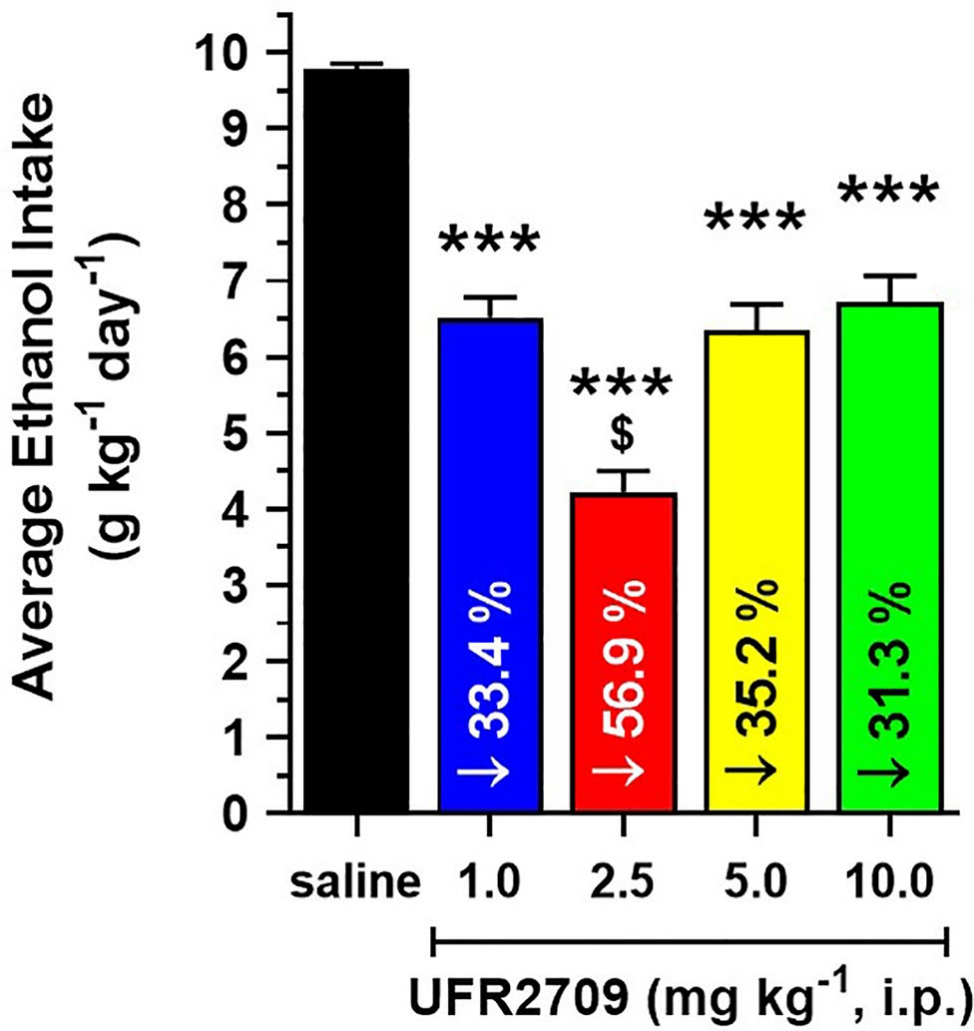
Average reduction in ethanol intake induced by 17 consecutive days of UFR2709 (1, 2.5, 5, 10 mg/kg/day i.p.) or saline (1 mL/kg i.p.) administration. Each bar represents the average ethanol intake of high-alcohol-drinking UChB rats (n = 5 per group) during the 17-day treatment period. Data are expressed as mean ± SEM (g/kg/day). One-way ANOVA followed by Tukey’s *post hoc* test was used to compare the saline and UFR2709 groups (***P < 0.001 vs. saline group; ^$^P < 0.001 vs. 2.5 mg/kg UFR2709 group). The number inside each bar indicates the percentage reduction in ethanol intake compared to the saline group.

**Figure 4:**
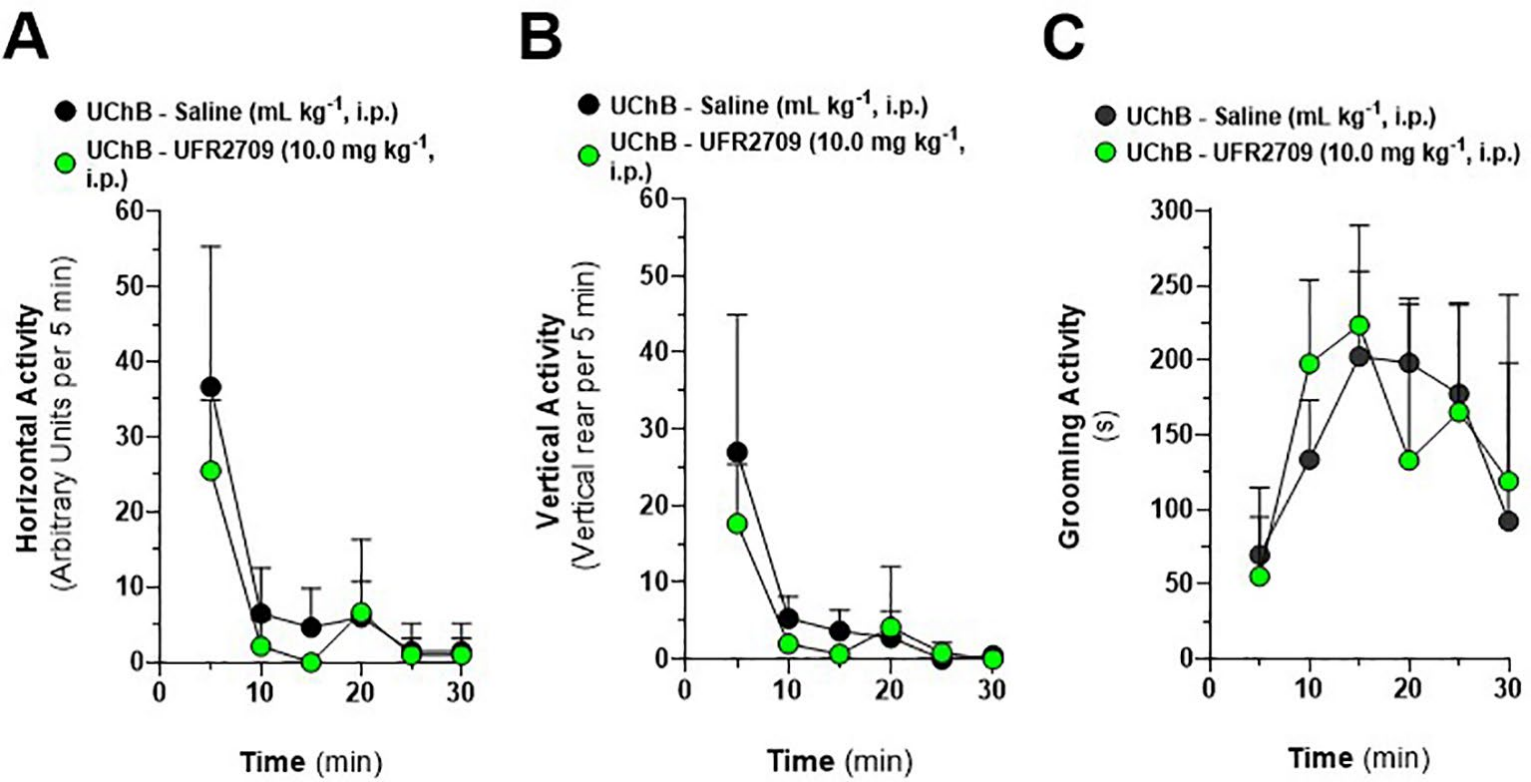
Effect of UFR2709 (10 mg/kg i.p.) or saline (1 mL/kg i.p.) administration on the locomotor activity and grooming behavior of UChB rats. Twelve rats were administered UFR2709 (n = 6) or saline (n = 6) 30 min before locomotor activity was assessed. **(A)** Time-course of horizontal activity per 5 min, presented as the number of arbitrary units (AU) of activity per 5 min. Results are expressed as the mean ± SEM. **(B)** Time-course of vertical activity per 5 min, presented as the number of vertical rears per 5 min. Results are expressed as the mean ± SEM. **(C)** Time-course of grooming activity per 5 min, presented as the time (s) of grooming activity per 5 min. Results are expressed as the mean ± SEM. Two-way ANOVA followed by Bonferroni’s *post hoc* test was used to compare the saline and UFR2709 groups.

Differences between UFR2709- and saline-treated animals were analyzed using two-way ANOVA with Tukey´s multiple comparison test (**Figures 1** and **2**). One-way ANOVA followed by Tukey’s *post hoc* test was used to analyze the effect of 17 days of saline or UFR2709 administration on average ethanol intake (**Figure 3**). The time-course of horizontal and vertical locomotor activity and grooming behavior was recorded every 5 min throughout the 30 min test period. Data were analyzed using two-way ANOVA followed by Bonferroni’s *post hoc* test to compare the effects of saline and UFR2709 (10 mg/kg, i.p.) (**Figure 4**). Data are expressed as mean ± SEM. Statistical analyses were performed using Graph Pad Prism 8.0 software (Graph Pad Software, San Diego, CA, USA), and the level of statistical significance was set at P < 0.05.

## Results

### Effect of UFR2709 on the Maintenance of Ethanol Intake by UChB Rats

To determine the effect of different doses of UFR2709 on the maintenance of voluntary ethanol intake, alcohol-preferring UChB rats were given a free choice between 10% ^v^/_v_ ethanol and water for 20 days. At day 20 of ethanol access, animals were administered a 1, 2.5, 5, or 10 mg/kg dose of UFR2709 or saline each day for 17 consecutive days. The baseline levels of ethanol consumption for all groups correspond to the average ethanol intake during the last 3 days before the treatment period. Two-way ANOVA with Tukey’s multiple comparison test with dose and day as factors showed that all UFR2709 doses significantly reduced ethanol intake (**Figure 1**) in comparison to saline (interaction [F_(76,400)_ = 2.992, P < 0.0001]; days [F_(19,400)_ = 10.20, P < 0.0001]; doses [F_(4,400)_ = 179.5, P < 0.0001]). In addition, all UFR2709 doses significantly increased water intake (**Figure 2**) in comparison to saline (interaction [F_(76,400)_ = 2.244, P < 0.0001]; days [F_(19,400)_ = 6.708, P < 0.0001]; doses [F_(4,400)_ = 77.82, P < 0.0001]).

**Figure 3** shows the total average ethanol intake across the treatment period of the groups administered different doses of UFR2709. One-way ANOVA indicated that UFR2709 treatment significantly reduced average ethanol intake [F_(4,80)_ = 50.18, P < 0.0001], and Tukey’s *post hoc* test confirmed that all UFR2709 doses significantly reduced average ethanol intake compared to saline. Administration of a 2.5 mg/kg dose of UFR2709 induced a 56.9% reduction in alcohol intake. All other UFR2709 doses induced smaller reductions in alcohol intake: 1, 5, and 10 mg/kg doses induced 33.4%, 35.2%, and 31.3% reductions, respectively. Administration of UFR2709 did not affect body weight compared to saline, and the rats exhibited normal increases in body weight during the course of this study (data shown in **Supplementary Material**).

### Effects of UFR2709 on Locomotor Activity

To determine if differences in alcohol consumption could be attributed to decreased locomotor activity, we assessed the locomotor activity of UFR2709- and saline-treated animals. For these experiments, animals were administered the highest dose of UFR2709 (10 mg/kg i.p) used in the aforementioned experiments. **Figure 4A** shows the time-course of horizontal locomotor activity measured every 5 min during the 30-min test period of UChB rats treated with UFR2709 (10 mg/kg) or saline. Two-way ANOVA showed that UFR2709 treatment did not affect locomotor activity compared to saline (treatment [F_(1,60)_ = 3.77, P = 0.057]). **Figure 4B** shows the time-course of vertical activity measured every 5 min during the 30-min test period of UChB rats treated with UFR2709 (10 mg/kg) or saline. Two-way ANOVA showed that UFR2709 treatment did not significantly affect the vertical activity of the animals (treatment [F_(1,60)_ = 2.47, P = 0.121]). **Figure 4C** shows the time-course of grooming activity measured every 5 min during the 30-min test period of UChB rats treated with UFR2709 (10 mg/kg) or saline. Two-way ANOVA showed that UFR2709 treatment did not significantly affect the grooming activity of the animals (treatment [F_(1,60)_ = 0.04, P = 0.845]).

### Distribution Coefficient

Prior to the experimental determination of Log D_7.4_, we theoretically calculated the cLogP value of UFR2709. The theoretical cLogP value for UFR2709 was 2.5, indicating that this drug should be able to access the central nervous system (CNS). As shown in **Table 1**, UFR2709 had an experimental LogD_7.4_ value of 1.14 ± 0.03 (assessed by the shake-flask method), which was higher than that obtained for nicotine (0.13 ± 0.01) (Zhu et al., 2002; Andrés et al., 2015), confirming its capacity to access the CNS. We also calculated the cLogP values of molecules capable of crossing the blood-brain barrier (BBB), namely imipramine, fluoxetine, methylphenidate, mecamylamine, and erysodine, obtaining values of 4.32, 4.27, 2.16, 2.38, and 1.40, respectively.

**Table 1:** Theoretical (cLogP) and experimental lipophilicity of UFR2709 and Nicotine were determined using the octanol-buffer distribution coefficient at pH 7.4 (Log D_7.4_).

Compound	cLogP	Log D_7.4_	Reference Log D_7.4_
UFR2709	2.15	1.14 ± 0.03.	–
Nicotine	0.93	0.13 ± 0.01	0.41^a^

Data represent the mean ± SEM of five experiments. ^a^Zhu et al., 2002.

## Discussion

Here, we report the effects of UFR2709, a non-selective competitive nAChR antagonist, on the ethanol consumption of high-alcohol-drinking UChB rats (Mardones and Segovia-Riquelme, 1983; Quintanilla et al., 2006; Tampier and Quintanilla, 2010). Our results show that all doses of UFR2709 tested elicited a reduction in voluntary ethanol consumption and a concomitant increase in water intake. However, the effect of UFR270 on reducing alcohol intake was bell-shaped—its efficacy increased from 1 mg/kg to 2.5 mg/kg and then reduced at higher concentrations (5 and 10 mg/kg). This gradual loss of effectiveness may be due to the development of tolerance, which we have previously observed with the partial agonists cytisine and varenicline in UChB rats (Sotomayor-Zárate et al., 2013).

The effects of UFR2709 on alcohol intake may have resulted from UFR2709 reducing locomotor activity *via* the inhibition of muscle nAChRs. This is unlikely, however, as administration of a single 10 mg/kg dose of UFR2709 to ethanol-naïve UChB rats had no effect on the time-course of horizontal, vertical, or grooming activity. Signs of discomfort or changes in body weight were not observed in the alcohol-exposed animals, suggesting that the effects of UFR2709 on alcohol intake were due to the inhibition of nAChRs in the mesocorticolimbic-DA system.

Our results support the view that nAChR inhibition in the mesocorticolimbic-DA system reduces ethanol consumption. Previous reports have indicated that systemic administration of mecamylamine, a non-competitive and non-selective nAChR antagonist that crosses the BBB (Bacher et al., 2009), reduces voluntary ethanol consumption in rodents (Blomqvist et al., 1996; Ford et al., 2009; Farook et al., 2009). Importantly, hexamethonium, a nAChR antagonist that does not cross the BBB, has no effect on ethanol intake (Blomqvist et al., 1996). Therefore, given that the Log D_7.4_ value of UFR2709 is between 1 and 3, which, according to the literature, is the optimum range for CNS penetration (Andrés et al., 2015), we suggest that it acts in the CNS. Furthermore, microdialysis experiments conducted in the striatum indicated that UFR2709 did not induce DA release, but rather elicited a slight decrease in basal DA levels, although this change was not statistically significant (data shown in **Supplementary Material**).

Even though UFR2709 displays higher affinity for α4β2 nAChRs than for α7 nAChRs (Faundez-Parraguez et al., 2013), the fact that ACh-induced currents are potentiated by ethanol (Aistrup et al., 1999; Cardoso et al., 1999) indicates that some other nAChR subtype(s) may be involved in the actions of UFR2709. Inhibition of α7 nAChRs does not modify the behavioral and neurochemical effects of ethanol (Larsson et al., 2002). However, α-conotoxin-MII, an antagonist of α3β2- and α6-containing nAChRs, blocks ethanol-associated conditioned reinforcement (Löf et al., 2007), and reduces ethanol-induced DA efflux and voluntary ethanol consumption in mice and rats (Larsson et al., 2004), suggesting that these nAChR subtypes may be involved in neurochemical effects of ethanol. Moreover, α6 subunit-containing nAChRs are predominantly expressed by dopaminergic neurons of the mesocorticolimbic-DA system (Klink et al., 2001; Champtiaux et al., 2002), and ethanol induces an α6 subunit-dependent increase in the firing rate of dopaminergic neurons in the VTA (Liu et al., 2013). Our study does not indicate the nAChR subtypes implicated in the effects of UFR2709, but clearly demonstrates that nAChR inhibition reduces ethanol intake. This supports the idea that nAChRs could be used as therapeutic targets for the treatment of alcohol abuse.

## Conclusion

In summary, UFR2709 reduces ethanol consumption in a dose-dependent manner. The effects of the highest doses of UFR2709 (5 and 10 mg/kg) were less sustained than those of the lower doses, suggesting that high doses induced drug tolerance. On the other hand, the 2.5 mg/kg dose of UFR2709 was the most potent and therapeutically effective, eliciting a long-lasting effect. Remarkably, this effect continued for at least 2 days after the last administration. Additionally, our results show that UFR2709 does not affect locomotor activity or body weight. Thus, our data give further support to the idea that UFR2709, a nAChR antagonist, may be a novel therapeutic agent for the treatment of alcoholism, and highlight nAChRs as potential targets for the design of drugs aimed to reduce ethanol intake.

## Data Availability Statement

All datasets generated for this study are included in the article/**Supplementary Material**.

## Ethics Statement

The animal study was reviewed and approved by University of Chile.

## Author Contributions

GQ, RS-Z, MQ, MR-P, MR-M, PI-V, and IB wrote the manuscript and they designed the experiments and interpreted the results. JG-G and PI-V synthesized UFR2709 and performed its characterization by ^1^H and ^13^C NMR analysis. RS-Z, MR-P, IB, and MR-M performed statistical analysis of data. GQ, MR-M, and MQ performed ethanol intake assays. MR-M and DL performed locomotor activity assays. All authors review critically the manuscript and GQ and RS-Z are considered the first authors of this work.

## Funding

This work was supported by “Fondo Nacional de Desarrollo Científico y Tecnológico” (FONDECYT) Grants 113-0012 (MQ), 117-0662 (MR-P), 115-0615 (PI-V), and 116-0398 (RS-Z). Additional funding was provided by PII2018 (MR-M). GQ and JG-G were fellows of the CONICYT National Ph.D. Scholar Fellowship.

## Conflict of Interest

The authors declare that the research was conducted in the absence of any commercial or financial relationships that could be construed as a potential conflict of interest.
