# Supplementary material for: UFR2709, a Nicotinic Acetylcholine Receptor Antagonist, Decreases Ethanol Intake in Alcohol-Preferring Rats
Source: Front Pharmacol. 2019 Dec 3;10:1429. doi: 10.3389/fphar.2019.01429 (PMC6901503; doi:10.3389/fphar.2019.01429)
Supplement: Supplementary file 1 [file DataSheet_1.docx]

***In vivo* brain microdialysis:** Adult UChB male rats (250–280 g) were deeply anesthetized with choral hydrate (400 mg/Kg, i.p.) and placed in a stereotaxic apparatus (model 68002, RWD Life Science Co. Ltd, Shenzhen, China). Body temperature of the animals was maintained at 37^◦^C with an electrical blanket controlled by a thermostat. Concentric brain microdialysis probes (2 mm membrane length, model CMA 11, 6,000 Daltons cut-off, Solna, Sweden) were implanted in Striatum using the coordinates according to the atlas of Paxinos and Watson. Microdialysis probes were perfused with Krebs-Ringer’s phosphate buffer (KRP in mM: NaCl 120; KCl 2.4; Na_2_HPO_4_ 0.9; NaH_2_PO_4_ 1.4; pH = 7.4) at a rate of 1 μL/min using an infusion pump (model RWD 210, RWD Life Science Co. Ltd, Shenzhen, China). All the dialysates were maintained on ice during the experiment and stored at -80^◦^C until analysis.

All experimental procedures were approved by the Ethics Committee of the Faculties of Science and Pharmacy at the Universidad de Valparaiso and the Institutional Animal Experimentation Ethics Board and the Science Council (FONDECYT) of Chile. Efforts were made to minimize the number of animals used and their suffering.

**Dopamine quantifications:** Ten microliters of each dialysate samples were injected to a HPLC system with electrochemical detector (set at 650 mV, 0.5 nA; model LC-4C, BAS, West Lafayette, IN, USA). Dopamine levels were assessed by comparing the respective peak area and elution time of the sample with a reference standard and the quantification was performed using a calibration curve (Program ChromPass, Jasco Co. Ltd., Tokyo, Japan).

**References**

[1] Paxinos G, Watson C. The rat brain in stereotaxic coordinates. 5th ed. Amsterdam ; Boston: Elsevier Academic Press. 2005.

**Figure S1:** Dopamine release in Striatum of adult male rats. Extracellular dopamine levels in the Striatum after systemic injection of nicotine (1 mg/Kg i.p.) or UFR2709 (10 mg/Kg i.p.). Asterisk indicates a significant difference when comparing the effect of drugs with respective baseline (#P<0.05, *P < 0.05, **P<0.01, ***P<0.001; one-way ANOVA followed by the Newman–Keuls multiple comparison test). Results are expressed as percentage of the respective basal levels (mean) ± SEM (n = 3 for each experimental group).

**Figure S2:** Body weight change of UChB rats treated with different doses of UFR2709 or saline.
